# A prevalence survey of enteral parasites in preschool children in the Mangochi District of Malawi

**DOI:** 10.1186/s12879-019-4439-8

**Published:** 2019-10-11

**Authors:** Timothy P. W. Jones, John D. Hart, Khumbo Kalua, Robin L. Bailey

**Affiliations:** 1 Department of Infectious Disease and Microbiology, Royal Free Hospital, Pond Street, London, NW3 2QG UK; 2 Clinical Research Department, London School of Hygiene and Tropical Medicine, Keppel Street, London, WC1E 7HT UK; 3 Department of Ophthalmology, University of Malawi, College of Medicine, Queen Elizabeth Central Hospital, P.O. Box E180, Blantyre, Malawi; 4 Blantyre Institute for Community Ophthalmology, Lions Sight First Eye Hospital, Queen Elizabeth Central Hospital, P.O. Box E180, Blantyre, Malawi

**Keywords:** Soil transmitted helminths, Growth restriction, Preschool children, Malawi, Mass drug administration

## Abstract

**Background:**

Helminthic and protozoan infections are common, particularly in low- or middle-income countries. Although an association between parasite carriage and markers of poor growth have been shown in some studies, systematic reviews have suggested only a modest impact of clearing carriage. The prevalence of these pathogens and the effect that they have on growth in preschool children has never been investigated in Malawi.

**Methods:**

One hundred ninety-three children aged 0–72 months were randomly recruited from rural villages in the Mangochi district of Malawi. Formol-ether concentration was performed on stool and the samples examined with a light microscope. Anthropometric data was taken for each child and the haemoglobin measured with a point of care test.

**Results:**

The mean age of the children was 2 years 4 months. Overall prevalence of intestinal parasite infection was 37.3%. Protozoa were found in 28.5% of children, while helminths were found in 8.8%. The most commonly found organisms were *Giardia lambia* (12.4%), *Entamoeba coli* (10.4%) and Hookworm species (3.6%). Stunting was seen in 47.8% of children, 12.9% were underweight and 5.0% were wasted. No significant association was found between markers of poor growth and infection with any intestinal parasite.

**Conclusions:**

We found that prevalence of helminth infection was low in preschool children living in the Mangochi district compared to international standards. However a significant proportion of the preschool population are infected with protozoa, particularly *Giardia lambia*. In this cohort, despite a significant prevalence of stunting, helminth infection was not significantly associated with any markers of poor growth. The significance of protozoal carriage and contribution to growth restriction in this context creates further avenues for future research.

## Background

Human Enteropathogens have been shown to exert a significant morbidity and mortality. Diarrhoeal diseases are the fifth leading cause of under 5 mortality worldwide, with the highest rates of death in Africa and Asia. Globally they account for greater loss of life in children under the age of 2 than HIV and malaria combined [1]. In 2007 a Malawian study suggested diarrhoeal diseases were sixth leading cause of mortality in paediatric patients [2]. The enteropathogens causing human disease are multiple, including parasites, bacteria and viruses. Diarrhoeal illness caused by *Cryptosporidium spp. and Entamoeba histolytica* have been shown to cause significant mortality in children under 5 years of age in low and middle income countries [3–5]. Studies suggest that diarrhoea is a poor marker of carriage; highlighting the need for asymptomatic prevalence figs [6].

Helminth infections have also long been held to cause a significant and serious public health problem in developing countries. Mass drug administration protocols from the WHO advocate treatment of communities where prevalence of soil-transmitted helminths is > 20%. These endeavour to target both direct effects on growth and development as well as indirect consequences of reduced vaccine efficacy and reduced immune response to pathogens [7, 8]. However, recent reviews of the impact of treatment by Cochrane and the Campbell collaboration have reported only weak evidence for weight gain and none on height gain after treatment [9, 10]. The effect on preschool children has been less extensively studied, but several papers have suggested some association [11].

Malawi also has high rates of poor growth amongst children, with the most recent national survey estimating that 37% of preschool children were stunted [12]. Malnourishment is associated not only with stunting and poor growth, but also with failure to reach cognitive potential in later life [2, 8]. The prevalence of soil-transmitted helminths in Malawi has not been extensively investigated, with the four studies [7, 13–15] conducted reporting variation in prevalence from 1.8% (13) to 77.4%. (7) Since these studies, the Malawian government has increased its coverage of mass drug administration for soil-transmitted helminths, with the aim to include children who were not attending school in 2012. No published study has measured prevalence of helminth carriage in preschool children in the country, while asymptomatic carriage prevalence of gut protozoa has not been examined in any children in Malawi.

We hypothesize that there is a significant burden of enteroparasites which may be exerting an effect on growth in this region. The aim of this study therefore, was to measure the prevalence of soil-transmitted helminths and intestinal protozoa burden in preschool children in the Mangochi district of Malawi, and to explore any association between carriage and growth restriction or anaemia.

## Methods

### Study design

This is a cross-sectional cluster survey study of the carriage of enteropathogens, in stool samples collected from children aged 1–72 months, in the Mangochi district of Malawi. The study took place between June and July 2016. Stool and blood samples were collected from children living in villages in the Mangochi District of Malawi, alongside the team working in the Childhood Mortality Reduction after Oral Azithromycin in Malawi (MORDOR-Malawi) study [16]. MORDOR-Malawi is a cluster-randomised placebo controlled trial exploring the effect of six-monthly administration of Azithromycin on under-five mortality, morbidity and growth.

### Study setting

**Fig. 1:**
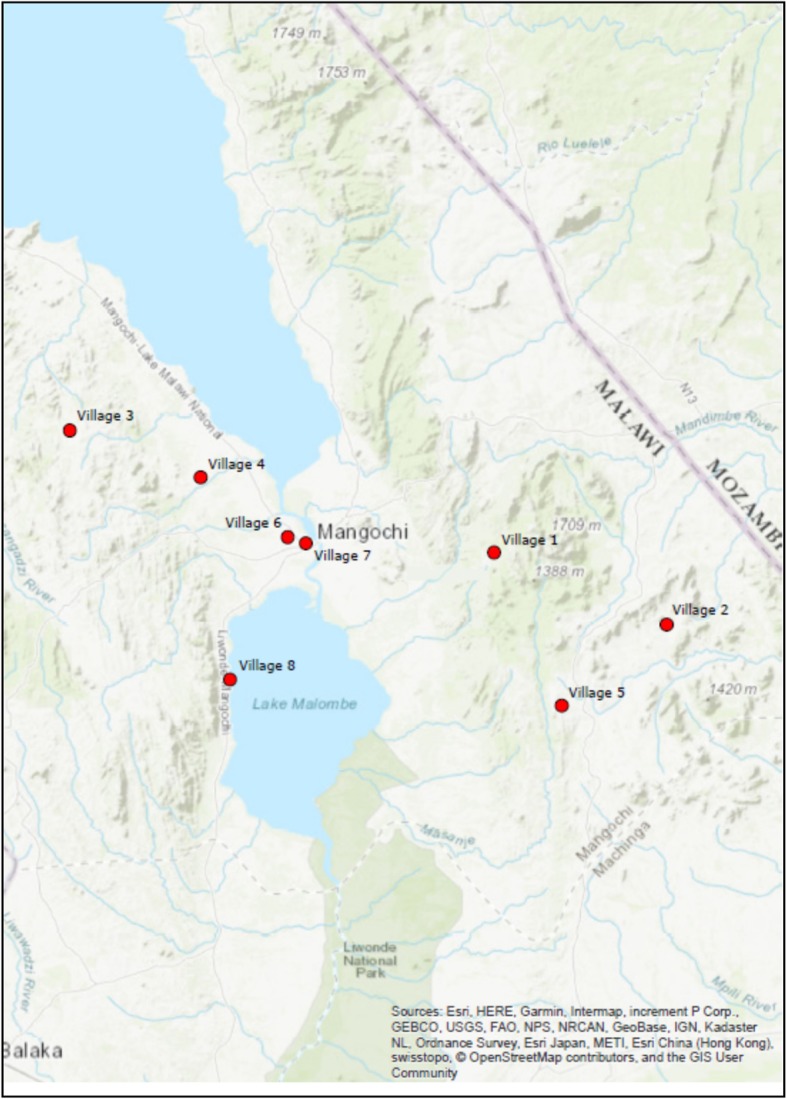
Map of Mangochi District of Malawi with location of sampled villages [17]

Mangochi is in the lakeside area of central Malawi, with a population on the most recent national census in 2008 of 610,239. It is situated 667 m above sea level at 14^o^ 24′ 35″ south, and 35^o^ 21′ 48″ east. It is less urbanised than other areas of Malawi, and has a greater proportion of people living in poverty and a higher under-five mortality rate. Within the district, 8 rural villages were used from areas close to Mangochi city and others close to the Mozambique border, the location of which are shown in Fig. 1 [17].

Children were recruited from households in the Mangochi District as part of the MORDOR-Malawi study [16]. Children were recruited to MORDOR-Malawi through the 2014 census of all children living in non-urban areas of the Mangochi District. Any child aged less than 60 months at the time was suitable for recruitment to MORDOR-Malawi. Additional entries and exits were allowed at each biannual census. Forty children were selected from the census using a computer based random number generator during the April to July treatment intervention of the MORDOR-Malawi study. The stool samples, blood samples and anthropomorphic data collected from these children were analysed in our study. Eight village clusters were used based upon availability of samples and data during the months of June and July 2016. The village status of Azithromycin or Placebo was unblinded only after completion of MORDOR-Malawi.

Consent forms were translated into the local language, and read aloud to the parent or guardian of every participant. Verbal and written consent were obtained. Details were collected on the child’s name, sex and date of birth with the use of health records cards.

Conservative estimates of a helminth prevalence of 8% were based upon previous studies in Malawi [7, 13, 15]. It was calculated that a sample size of 179 would be sufficient to estimate prevalence. This was calculated using n = (Z^2^P(1-P))/d^2^ where Z = Z statistic for confidence (1.96 for 95%), P = expected prevalence (8.1%), d = precision (4%); recommended to be half of P in cases where *P* < 10% [18]. This was corroborated by WHO practice which advises samples of 200 are sufficient in ecologically homogeneous areas.

### Outcome measures

Prevalence of enteropathogen parasite infection was assessed by identification of cysts or ova on light microscopy in faecal samples. All species of gut helminths and protozoa were assessed for.

Anthropometric data was collected for height, weight and middle upper arm circumference (MUAC) of the individuals. Prevalence of being underweight, stunting and wasting were recorded based on the age and gender-specific z-scores. These were calculated using the WHO AnthroPlus software (WHO, Geneva, Switzerland 2007). A height for age z-score (HAZ) below 2 standard deviations was defined as stunting, a weight for age z-score (WAZ) below 2 standard deviations was defined as being underweight and weight-for-height z-score (WHZ) below 2 standard deviations was defined as wasting. Haemoglobin was measured in grams per decilitre, recorded using a point of care machine.

### Study procedures

A clean single use 50 ml screw top container for the collection of faecal samples was provided to the parents of participants, along with an oral explanation and written information on how to obtain samples. Samples were stored in cool boxes, collected on the same day or the following morning and transported from the field to the MORDOR-Malawi Lab in Mangochi on ice. Samples were processed on arrival at the lab and were read within 24 h of processing.

The collected stool samples were concentrated using the formol-ether method to allow for identification of protozoa and helminth species. Two drops of suspended solution were placed on a microscope slide, one mixed with Lugol’s Iodine and examined under high power microscope by a single trained microscopist for size, and features of helminth or protozoa cysts and ova. 5% of samples were independently checked by a second trained microscopist. Microscopists were trained in the identification of all regional human helminths; *Ascaris lumbrocoides,* Hookworm sp.*, Trichuris trichiura, Schistosoma mansoni, Strongyloides stercoralis, Taenia sp. Hymenolepis nana.* As well as important human enteral protozoa; *Entamoeba histolytica, Entamoeba hartmani, Entamoeba coli, Iodamoeba butschlii, Endolimax nana, Giardia sp., Chilomastix mesnili, Dientamoeba fragalis, Cryptosporidium parvum, Cylocspora cayetanensis, Isospora belli.*

A trained nurse collected blood Microcuvettes and analysed for haemoglobin concentration using a Hemocue analyser (Hemocue AB, Ängelholm, Sweden). The results were anonymously recorded on a database produced for the purpose.

Trained anthropometrists recorded height, weight, MUAC and head circumference of all the children. Measurements were taken in triplicate, with the largest measurement used for analysis. Height was assessed with a ShorrBoard (ShorrBoard®, Shorr Productions, LLC, Olney, MD, USA) measuring to 1 mm, weight with an electronic scale (Seca 874/878 flat floor scale, Seca GMBH & Co. Kg, Hamburg, Germany) correct to 0.1 kg. MUAC and head circumference were measured to the nearest millimetre using non-stretch MUAC tapes. Z-scores were calculated using the WHO AnthroPlus software (WHO, Geneva, Switzerland 2007).

### Statistical analysis

Data collected from the study were stored in Microsoft access 2016, exported to Microsoft excel 2016 and analysed using STATA 14.1. (StataCorp LP, Texas, USA).

Quantitative variables were summarised as means, and the standard deviations were calculated. Data was expressed as proportions to assess the prevalence of gastrointestinal parasite carriage and stunting, wasting and being underweight. Proportions were compared using a chi-squared test and Fishers exact test where this was appropriate. Student t-test was used for comparison of means.

Sensitivity analysis was performed between villages treated with Azithromycin and placebo to investigate for any measurable impact on carriage of enteropathogens or growth. Chi Squared test was used to compare for difference in the proportion of infected children in each of the two treatment arms, while ANOVA was used to assess for differences in mean growth markers.

### Ethical approval

Ethical approval was granted prior to study commencement by the London School of Hygiene MSc Research Ethics Committee (Ref: 10872). Local ethical approval was obtained as part of the MORDOR – Malawi study by the College of Medicine Research and Ethics Committee (COMREC) in Malawi (Ref: P.02/14/1521). Further details can be found in the declarations section below.

## Results

### The sample demographics

**Fig. 2:**
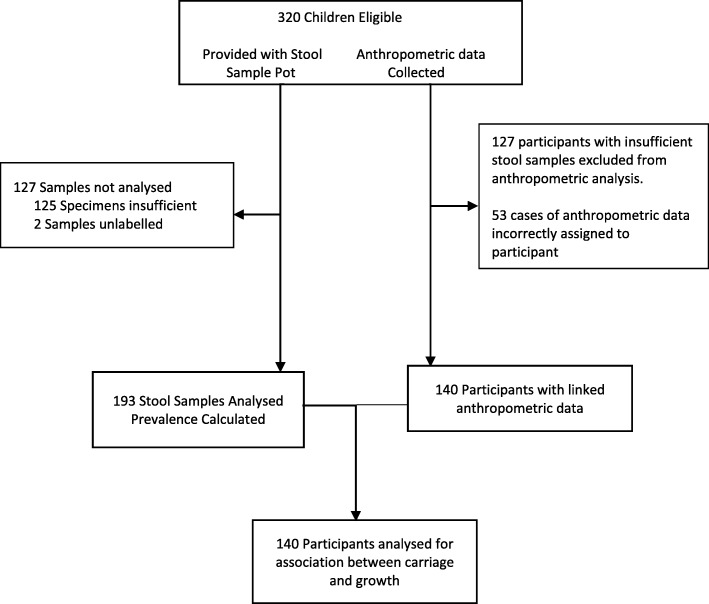
Enrolment of participants and analysis of data

A total of 320 children were recruited for the study from 8 villages and provided with sample bottle for stool specimens. Of the 320 children recruited a total of 219 samples were returned. Demographic and anthropometric data and demographic data was collected for all children, however in 53 cases this data was not linked due to incorrect assignment of blinded anthropometric patient identification numbers. Fig. 2 provides a flow chart of study recruitment.

Slightly over half of the children were female (52.8%). The mean age of the children was 2.35 years (range 3 months to 5 years 6 months). Mean age of boys was 2.8 years, and girls 2.7 years. The demographics of the cohort are displayed in Table 1.

**Table 1:** Demographic data of study particpants

			WEIGHT (kg)	HEIGHT (cm)	HC (cm)
Male	67/140	(47.8%)	12.2	85.5	47.9
Female	73/140	(52.2%)	11.6	85.0	47.5
Age (y)					
0–1	17/140	(12.1%)	7.4	65.8	43.8
1–2	22/140	(15.7%)	9.5	76.6	45.8
2–3	36/140	(25.7%)	11.9	84.2	48.0
3–4	32/140	(22.9%)	13.2	90.4	48.5
4–5	26/140	(18.6%)	14.1	95.7	49.8
5–6	7/140	(5.0%)	16.7	101.9	50.2
Weight for Age (z-score)		Mean		−0.87 (S.D 1.17)	
		Mean Male		−0.85 (S.D 1.08)	
		Mean Female		−0.89 (S.D 1.24)	
		Minimum		−5.24	
		Maximum		1.81	
Height for Age (z-score)		Mean		−1.80 (S.D 1.88)	
		Mean Male		−1.96 (S.D 1.78)	
		Mean Female		− 1.66 (S.D 1.96)	
		Minimum		−5.74	
		Maximum		4.56	
Weight for Height (z-score)		Mean		0.19 (S.D 1.36)	
		Mean Male		0.39 (S.D 1.35)	
		Mean Female		0.03 (S.D 1.36)	
		Minimum		−5.03	
		Maximum		3.81	
Haemoglobin (g/dL)		Mean		10.8 (S.D 1.61)	
		Mean Male		10.7 (S.D 1.71)	
		Mean Female		10.97 (S.D 1.52)	
		Minimum		7	
		Maximum		14.3	

All averages expressed as mean. *HC* Head Circumference

### Prevalence of infection

A total of 193 stool samples were collected, 72 (37.3%) were infected with Intestinal parasites. Protozoal infection (*n* = 55 (28.5%)) was more prevalent than helminths (*n* = 17 (8.8%)). *Giardia. lambia* was the most prevalent protozoal species with 12.4% of samples containing cysts, followed by *Entamoeba. coli* with a prevalence of 10.4%. *Entamoeba. histolytica/dispar*, *Entamoeba. hartmanni* and *Iodamoeba butschlii* were also found, the prevalence for which are shown in Table 2. Hookworm species was the most prevalent helminth species with 3.6% of the population being found to be positive, followed by *Taenia species*. Other helminths found included *Hymenolepis species* [2] *Ascaris. lumbricoides* [2], *Trichuris trichiura* [1] *Strongyloides stercoralis* [1]. Prevalence of infection varied between different villages from 14.3 to 47.1%. No association was found between azithromycin administration and prevalence of helminth (*p* = 0.776) or enteral protozoal (*p* = 0.113) infection.

**Table 2:** Prevalence of cases of intestinal parasite infection in study participants seperated by village

	Total		Village 1	Village 2	Village 3	Village 4	Village 5	Village 6	Village 7	Village 8
Parasite	*n = 190*		*n = 32*	*n = 14*	*n = 12*	*n = 19*	*n = 2*	*n = 21*	*n = 17*	*n = 19*
Hookworm sp.	7	(3.6%)	(6.3%)	(0.0%)	(8.3%)	(0.0%)	(0.0%)	(0.0%)	(11.0%)	(0.0%)
Taenia sp.	4	(2.1%)	(0.0%)	(0.0%)	(8.3%)	(0.0%)	(0.0%)	(4.8%)	(0.0%)	(0.0%)
A. lumbricoides	2	(1.0%)	(0.0%)	(7.1%)	(8.3%)	(0.0%)	(0.0%)	(0.0%)	(0.0%)	(0.0%)
Hymenolepis sp.	2	(1.0%)	(3.1%)	(0.0%)	(0.0%)	(0.0%)	(0.0%)	(0.0%)	(0.0%)	(0.0%)
T. trichura	1	(0.5%)	(0.0%)	(0.0%)	(0.0%)	(0.0%)	(0.0%)	(4.8%)	(0.0%)	(0.0%)
Strongyloides	1	(0.5%)	(0.0%)	(0.0%)	(0.0%)	(0.0%)	(0.0%)	(0.0%)	(0.0%)	(0.0%)
Any Helminth	17	(8.8%)	(9.4%)	(7.1%)	(16.7%)	(0.0%)	(0.0%)	(14.3%)	(11.8%)	(0.0%)
*G. lamblia*	24	(12.4%)	(9.4%)	(7.1%)	(0.0%)	(21.1%)	(0.4%)	(19.0%)	(5.9%)	(25.0%)
*E. coli*	20	(10.4%)	(12.5%)	(0.0%)	(16.7%)	(15.8%)	(0.0%)	(14.3%)	(23.5%)	(6.3%)
E. histolytica/dispar	7	(3.6%)	(6.3%)	(0.0%)	(0.0%)	(0.0%)	(0.0%)	(0.0%)	(0.0%)	(12.5%)
*E. hartmanni*	3	(1.6%)	(0.0%)	(0.0%)	(0.0%)	(5.3%)	(0.0%)	(0.0%)	(5.8%)	(6.3%)
I. butschlii	1	(0.5%)	(0.0%)	(0.0%)	(0.0%)	(0.0%)	(0.0%)	(4.8%)	(0.0%)	(0.0%)
Any Protozoa	55	(28.5%)	(18.8%)	(7.1%)	(16.7%)	(36.8%)	(0.0%)	(33.3%)	(35.3%)	(43.8%)
Total	72	(37.3%)	(31.3%)	(14.3%)	(33.3%)	(36.8%)	(0.0%)	(47.6%)	(47.1%)	(43.8%)

There was an increase in the odds of being infected by protozoa in 4 and 5 years olds compared to those under one year of age (OR 5.5 [0.92–32.76] *p* = 0.04 and 10.0 [0.92–32.76] *p* = 0.02 respectively). When a chi-squared test for trend was calculated, as age increased and increased odds of infection by any parasite or any protozoa was found. Table 3 reports the full set of prevalence and odds ratios of infection by any parasite, any helminth and any protozoa stratified by age group.

**Table 3:** Prevalence and odds ratio of Infection with parasite, helminth or protozoa by age

Age	Total	ANY PARASITE			ANY HELMINTH			ANY PROTOZOA		
(years)		*% (n)*	*OR [95% CI]*	*p*	*% (n)*	*OR [95% CI]*	*p*	*% (n)*	*OR [95% CI]*	*p*
0–1	17	17.7 (3)	1.00	–	5.9 (1)	1.00	–	11.8 (2)	1.00	–
1–2	22	27.3 (6)	1.75 [0.35–8.59]	0.48	4.6 (1)	0.76 [0.04–13.64]	0.85	22.7 (5)	2.21 [0.35–13.64]	0.38
2–3	36	36.1 (13)	2.63 [0.61–11.35]	0.17	16.7 (6)	3.20 [0.33–30.31]	0.28	19.4 (7)	1.81 [0.32–10.06]	0.49
3–4	32	34.3 [11]	2.44 [0.55–10.77]	0.22	9.4 (3)	1.65 [0.15–17.75]	0.87	25.0 (8)	2.50 [0.44–13.92]	0.27
4–5	26	46.2 (12)	4.00 [0.84–18.86]	0.06	3.9 (1)	0.64 [0.03–11.38]	0.75	42.3 (11)	5.50 [0.92–32.76]	0.04
5–6	7	57.1 (4)	6.22 [0.71–54.29]	0.06	0 (0)	–	–	57.1 (4)	10.0 [0.87–114.9]	0.02
Total	140	49	Trend *p*= 0.024		12	Trend *p*= 0.630		37	Trend *p* = 0.006	

Groups stratified by age. Chi squared test use to calculate *p* values and for test of trend

On separate analysis there was also a significantly lower chance of being infected with any protozoa in children aged less than 36 months (OR 0.41, 95% CI [0.19–0.90] *p* = 0.02), compared to those aged 37 to 72 months. When individual pathogens were assessed, only the presence of *G. lambia* infection was found to be associated with a significantly reduced odds of infection in those aged less than 36 months (OR 0.29, 95% CI [0.10–0.85] *p* = 0.02). There was no significant difference in the prevalence of infection between the genders in any category of infection.

### Nutritional status

Stunting was found at an overall prevalence of 47.8% amongst the children, while being underweight was less common occurring in 12.9% of the children examined. Wasting was rare; only 7 (5.00%) of the children met criteria for wasting, all of which occurred in children aged under 3. Severe acute malnutrition (weight-for-height > 3 S. D below mean z score) was seen in only 2 (1.42%) children. Further breakdown by age of the prevalence of markers for poor nutritional status are displayed in Table 4. No statistically significant difference between mean WAZ (*p* = 0.194), HAZ (*p* = 0.08) or WHZ (*p* = 0.753) were found between villages treated with Azithromycin and those treated with placebo.

**Table 4:** Prevalence and odds ratio of growth restriction in patient cohort

	Underweight				Stunting				Wasting			
Age (Years)	*N*	%	OR [95% CI]	*p*	*N*	%	OR [95% CI]	*p*	*N*	%	OR [95% CI]	*p*
0–1	2/17	11.1	1	–	6/17	35.3	1	–	3/17	17.7	1	–
1–2	5/22	22.7	2.21 [0.35–13.65]	0.38	12/22	54.6	2.20 [0.57–8.43]	0.24	3/22	13.6	0.74 [0.13–4.31]	0.73
2–3	4/36	11.1	0.94 [0.10–5.80]	0.94	18/36	50.0	1.83 [0.55–6.17]	0.32	1/36	2.8	0.13 [0.01–1.55]	0.06
3–4	4/32	22.2	1.07 [0.17–6.67]	0.94	16/32	50.0	1.83 [0.53–6.32]	0.33	0/32	0.0	0	–
4–5	3/26	12.5	0.98 [0.14–6.71]	0.98	12/26	46.2	1.57 [0.44–5.66]	0.49	0/26	0.0	0	–
5–6	0/7	0.0	0	–	1/7	14.3	1.38 [0.22–8.66]	0.73	0/7	0.0	0	–
Total	18/140	12.9			67/140	47.8			7/140	5.0		

Odds Ratio with 95% confidence intervals of underweight, stunting and wasting as stratified by age group

### Relationship between nutritional status and parasitic infection

Odds ratios and Chi-Squared test was performed to examine the association between infection and markers of poor nutritional status. No significant association was found between infection and markers of poor childhood nutrition. (Table 5).

**Table 5:** Prevalence and odds ratio of growth restriction by parasite carriage

	Underweight				Stunting				Wasting			
	*n*	%	OR [95% CI]	*p (Χ*^2^)	*N*	%	OR [95% CI]	*p (Χ*^2^)	*n*	%	OR [95% CI]	*p (Χ*^2^)
Any Parasite												
*Absent*	14/91	15.4	1	0.223	45/91	49.5	1		5/91	5.4	1	
*Present*	4/49	8.2	0.49 [0.15–1.59]		22/49	44.9	0.83 [0.41–1.68]	0.607	2/49	4.1	0.73 [0.13–3.95]	0.714
Any Helminth												
*absent*	18/128	14.1	1		62/128	48.4	1		6/128	4.7	1	
*Present*	0/12	0	–	–	5/12	41.7	0.76 [0.22–2.53]	0.653	1/12	8.3	[0.20–16.94]	0.580
Any Protozoa												
*absent*	14/103	13.6	1		50/103	48.5	1		6/103	5.8	1	
*Present*	4/37	10.8	0.77 [0.24–2.52]	0.665	17/37	46.0	0.90 [0.42–1.92]	0.786	1/37	2.7	0.45 [0.05–3.91]	0.455

Odds ratio with 95% confidence intervals of underweight, stunting and wasting as presence of absence of enteroparasite. *P* value calculated from Chi-squared

Similarly, association between haemoglobin and infection status was performed. The mean haemoglobin was 10.84 g/dL. No association was found between infected individuals and haemoglobin.

## Discussion

This study found a low prevalence of helminthic infection in preschool children compared to international guidelines from the WHO [19]. Hookworm.sp. was the most common species, with a prevalence of 3.64% while *Giardia. lambia* was the most common protozoa with a prevalence of 12.44%.

The finding of a low prevalence of helminthic infection in preschool children in this region of Malawi would be in line with the more recent epidemiological surveys that have been performed on school aged children, which reported a prevalence of soil-transmitted helminths of just 1.8% [13]. This is significantly less than early studies published by Phiri and Randell reporting prevalence as high as 60% [7, 15]. There were some differences between this study and the previous Malawian papers. The protocol chosen for this study was that of formol ether concentration. This was employed to allow the identification of protozoa, something which was not achieved in any of the three previous large studies which all used Kato Katz. Kato-Katz is the method favoured by the WHO in prevalence studies [8], however formol ether concentration holds up well against Kato Katz in previous analysis [20, 21]. In one direct comparative study formol ether showed a significantly improved sensitivity for *Ascaris,* Hookworm and *Trichuris* (58.3, 69.5 and 88.5%) when compared to a single Kato Katz slide (38.9, 39 and 85%) [21]. A single slide obtained from a single stool sample was the method preferred in the two previous helminth studies in Malawi. Therefore, from a methodological point of view the results obtained in this study are comparable. The nature of helminthic and protozoal infections is that ova and cysts are shed only intermittently. As such it is recommended that 3 samples are collected and examined. This has been shown to significantly increase the sensitivity up to 99.9% for *Ascaris* [22]. There was not sufficient time in this study to achieve this; it is therefore likely that the true prevalence is higher than suggested here. Hookworm eggs are also particularly sensitive to degradation, although samples were refrigerated to prevent hatching and fixed with formol-ether on arrival, it is possible that this time delay may have contributed to the low prevalence of this particular helminth.

Reasons for the significantly lower figures for helminths infection rate in this Malawian study are unclear. The systematic targeting of preschool children by the deworming programmes is more difficult than school aged children, but is normally organised in health clinics. School aged children have been shown to be the most heavily infected [23]. It is also known that there is a clear association between high prevalence and high intensity infection in these children [24]. Therefore, effective and widespread control programs could be one reason for the low prevalence seen in this study. In Malawi there is incomplete data on the availability of soil transmitted helminths treatment in preschool children. The ‘NTD Master Plan’ set out by the Malawian Ministry of Health sets out an aim to treat at least 75% of school aged children by 2020. No such aims exist in the country for preschool children, where treatment programs are not uniform and have relied upon NGO and global partners. The national Schistosomiasis Control Programme and Community Health Services unit does not publish details of where treatment has been offered. As part of the Lymphatic Filariasis national program all school aged children in the district were treated with albendazole and Ivermectin between 2009 and 2013, reporting over 75% coverage (personal correspondence; S. Jemu, Ministry of Health, Lilongwe, Malawi. February 2019). Furthermore previous studies have also reported personal correspondence suggesting that high risk villages are receiving treatment [13]. It is therefore difficult to comment with certainty on the role played by current deworming programs in the specific villages studied, although it remains likely that this has been important in reducing helminth carriage.

Similarly, national drives towards the use of soap and sanitation have improved the percentage of households with soap or other cleaning agents to 56.2% in 2015. However localised data regarding Mangochi district is not readily available. The same report states that the percentage of stools safely disposed of for children under the age of two years is 88.2% and the number of people using unimproved sanitation was reduced from 67.2 to 32.5% since 2006 [25]. It therefore seems credible that this improvement in sanitation too might contribute to the low prevalence of helminthic infections found.

There was marked variation in the prevalence of enteroparasite carriage between villages; as high as 47.1% positive in one community and as low as 14.3% in another. In order for a population to maintain an infection, it requires a large enough pool of infected individuals. In the case of helminths, where there is no zoonotic host, it follows that infections will increasingly be clustered in local communities and it is important to survey many distinct communities within an area to get an accurate regional prevalence. It is a strength of this study that many different geographically isolated communities over the district were investigated to give a more holistic picture of the true average prevalence in Mangochi. This did not appear to be the case in the national survey conducted by Randall, where just 5 schools were selected from each of the six ‘ecological regions’ into which the country had been divided [7].

Protozoal infections were more common than those of helminths, a total of 28.5% were found to be carrying at least one organism. There has been no previous study to compare our findings with in Malawian children. A study in adults revealed a lower prevalence of *Giardia* (1.1%) [14], but studies in other geographic areas have shown that preschool children have a higher incidence of protozoal infections [26].

There was a significant proportion of children meeting the criteria for poor growth. It is notable that over 40% of children were found to be stunted. Elucidating the causal factors is not straightforward. Many factors have been associated with stunting and other markers of poor growth both in utero and after birth. The initial 1000 days from conception is highlighted as the most important periods. Inadequate maternal and child macro- or micro-nutrition, preterm birth, lack of clean water and sanitation and recurrent infections in early childhood, particularly diarrhoeal diseases, have all been shown to exert an effect. Many of these factors are interrelated [3, 27]. The role of helminths in impairing child growth is less clear, with limited impact of mass drug administration strategies in meta-analysis studies [9]. Our study did not find an association between parasitic infection of any kind and the markers of stunting, wasting or being underweight. This might be explained by the low prevalence of most helminths, suggestive of a greater proportion of low intensity infections a finding supported in other countries [28, 29]. There remains debate in the literature over the association of *Giardia* and growth restriction with several groups finding an association [30–32], while others have not [33–35]. It is postulated certain strains may confer greater pathology [34, 36], with diarrheal losses thought to be the driving force behind the growth failure seen in *Giardia* infections [34, 37, 38]. As we did not enquire regarding diarrhoeal symptoms, it is not possible to differentiate symptomatic and asymptomatic cases in this study.

There were some weaknesses to our study; we did not attempt to evaluate the diet or intake of iron of the participants, and it was not possible to obtain the malaria status of subjects. These may have impacted on the growth of children and haemoglobin levels. Confounding factors may also explain the lack of effect on growth parameters seen in this study. Malawi had also experienced at least two years of poor harvest due to flooding and drought, leading to significant food insecurity. We did not collect information on the socioeconomic status of families as this was not felt to be significantly variable in the rural communities assessed, however this may have compounded availability of good nutritional intake for some children. This study did not sample urine for *Schistosomiasis haematobium* which has been associated with stunting in preschool children [39], there is also evidence for association between maternal genitourinary schistosomiasis infection and low birth weight [40]. Although local prevalence levels are not available in either of these groups, it remains credible in this geographic environment that this may also be impacting growth both in utero and in the early years of life. As discussed above it was not possible to confirm the recent availability of de-worming medication in these communities, which may account for some of the variation in parasite prevalence. Some of the children in this study were in an Azithromycin arm of the MORDOR-Malawi study. It has been suggested that antibiotics may have growth promoting effects [41]. The significance of a single dose every six months is less clear and currently under investigation. From our own analysis there did not appear to be a significant effect on growth or worm burden.

Due to time constraints it was not possible to test more than one stool sample for each participant and, this may have impacted our sensitivity, especially as it appears that local prevalence is low. We attempted to reduce the error associated with a single sample by using a single formol ether concentration examination, which has been shown to have similar sensitivity to multiple Kato-Katz specimens [21]. It is likely that our study underestimates the true prevalence, especially in the case of Hookworm *sp.*; however it must be noted that all previous published studies in Malawi have also had this weakness, making the context of our prevalence comparable.

There was a significant discrepancy between the number of stool specimens investigated and matching anthropometric, and demographic data. Although complete data sets were taken, some samples were incompletely logged and not matched to the children surveyed. This limits the power of this part of the study, and highlights the difficulties experienced when conducting large complex studies in a blinded manner. A greater redundancy in the number of recruited individuals is required to prevent this from occurring. A larger sample of complete data, increasing the power, would have reduced the size of the confidence intervals, and ultimately could have revealed a statistically significant correlations.

## Conclusions

This study suggests that the prevalence of helminthic worms in the Mangochi District of Malawi is low. For the first time in Malawi we have quantified the prevalence of enteral protozoa in preschool children. No association was found between parasite carriage and growth restriction. Further investigation is warranted in the region where there are facilities to use diagnostic procedures with greater sensitivity, to further assess the factors responsible for continued poor child growth in the community.

## Data Availability

The datasets used and analysed during the current study are available from the corresponding author on reasonable request.

## Abbreviations

HAZ: Height for age z-score
MUAC: Middle Upper Arm Circumference
WAZ: Weight for age z-score
WHZ: Weight-for-height z-score
